# Bone Mineral Density Compared to Trabecular Bone Score in Primary Hyperparathyroidism

**DOI:** 10.3390/jcm11020330

**Published:** 2022-01-10

**Authors:** Alicia R. Jones, Koen Simons, Susan Harvey, Vivian Grill

**Affiliations:** 1 Department of Endocrinology and Diabetes, Western Health, Furlong Road, St Albans, VIC 3021, Australia; Vivian.grill@wh.org.au; 2 Centre for Epidemiology and Biostatistics, Melbourne School of Population and Global Health, The University of Melbourne, Bouverie Street, Melbourne, VIC 3010, Australia; koen.simons@unimelb.edu.au; 3 Office for Research, Western Health, Furlong Road, St Albans, VIC 3021, Australia; 4 Department of Medicine—Western Campus, The University of Melbourne, Furlong Road, St Albans, VIC 3021, Australia; harveys@unimelb.edu.au

**Keywords:** hyperparathyroidism, osteoporosis, bone mineral density, fractures

## Abstract

Individuals with primary hyperparathyroidism (PHPT) have reduced bone mineral density (BMD) according to dual X-ray absorptiometry at cortical sites, with relative sparing of trabecular BMD. However, fracture risk is increased at all sites. Trabecular bone score (TBS) may more accurately describe their bone quality and fracture risk. This study compared how BMD and TBS describe bone quality in PHPT. We conducted a retrospective cross-sectional study with a longitudinal component, of adults with PHPT, admitted to a tertiary hospital in Australia over ten years. The primary outcome was the TBS at the lumbar spine, compared to BMD, to describe bone quality and predict fractures. Secondary outcomes compared changes in TBS after parathyroidectomy. Of 68 included individuals, the mean age was 65.3 years, and 79% were female. Mean ± SD T-scores were −1.51 ± 1.63 at lumbar spine and mean TBS was 1.19 ± 0.12. Only 20.6% of individuals had lumbar spine BMD indicative of osteoporosis, while 57.4% of TBS were ≤1.20, indicating degraded architecture. There was a trend towards improved fracture prediction using TBS compared to BMD which did not reach statistical significance. Comparison of 15 individuals following parathyroidectomy showed no improvement in TBS.

## 1. Introduction

The clinical picture of primary hyperparathyroidism (PHPT) has evolved over the past century, from a classic symptomatic presentation involving ‘bones, stones, and psychic moans’ to a predominantly asymptomatic condition detected on biochemical screening [1]. While the overt skeletal condition of osteitis fibrosa cystica has become rare in developed countries, even mild, ‘asymptomatic’ PHPT has detrimental effects on the skeleton [2,3]. Early studies of bone mineral density (BMD) by dual X-ray absorptiometry (DXA) in PHPT demonstrated preferential reduction of BMD at sites with predominantly cortical bone such as the radius, with relative sparing of sites with predominantly trabecular bone such as the lumbar spine (LS) [4,5]. This pattern was also described by histomorphometry from iliac crest biopsies, which demonstrated preservation and even an increase in trabecular bone volume in PHPT [6].

Despite the preserved BMD at the LS by DXA, individuals with PHPT have an increased incidence of both vertebral fractures (VF) and non-vertebral fractures [7,8,9]. Parathyroid hormone (PTH) increases both bone resorption and formation. However, when continuously elevated as in PHPT, bone resorption predominates, with a catabolic effect on the skeleton [3]. Although PHPT predominantly affects post-menopausal women, who have an increased risk of osteoporosis and fractures due to ageing and loss of the protective effects of oestrogen, PHPT further increases this risk.

The paradox between increased VF despite preserved LS BMD suggests that areal BMD fails to capture all determinants of bone strength. Newer imaging modalities that examine bone microstructure have provided some insights into the discrepancy. High-resolution peripheral quantitative computed tomography (HRpQCT), a non-invasive technique that assesses trabecular and cortical microstructure at the distal radius and tibia, has shown abnormalities at both trabecular and cortical sites in postmenopausal women with PHPT [10,11,12]. Unfortunately, HRpQCT is expensive and not widely available for clinical use.

Trabecular bone score (TBS) is an index of grey-level texture variation obtained from LS DXA images [13]. Using TBS software as an addition to standard DXA, the same region can be analysed for BMD and TBS, with no additional imaging, radiation, or cost to the individual.

TBS has been shown to predict fractures in post-menopausal women [14,15,16] and older men [17], independent of BMD, and can improve fracture prediction in individuals with normal or osteopenic BMD [14,18]. TBS can be factored into the Fracture Risk Assessment Tool (FRAX) to improve fracture risk prediction. The value of TBS has also been examined in cases of secondary osteoporosis, such as glucocorticoid-induced osteoporosis [19], diabetes [20], chronic obstructive pulmonary disease [21], and end stage kidney disease [22].

In adults with PHPT, a good correlation between TBS and HRpQCT has been found [23]. TBS has been shown to be correlated with VF in post-menopausal women and in older men with PHPT, independent of LS BMD, with less evidence for correlation with non-vertebral or total fractures [24,25]. Studies examining changes in TBS after parathyroidectomy are conflicting, with some showing improvement in TBS [25,26,27,28] and others showing no change [29,30,31], which may be related to differences in the population studied, disease severity, or sample size.

The aims of this study were to examine the TBS together with areal BMD by DXA in an Australian cohort with PHPT, to assess the value of TBS compared to BMD in predicting prevalent fractures in this cohort, and to analyse changes in TBS after surgical cure by parathyroidectomy. We hypothesised that a greater proportion of patients would have abnormal TBS compared to abnormal BMD T-scores, and that TBS would be more strongly correlated to prevalent fractures than BMD.

## 2. Materials and Methods

This was a retrospective study at a tertiary teaching hospital in Victoria, Australia. The study was approved by the hospital’s Human Research Ethics Panel (QA2017.52).

### 2.1. Participants and Setting

Patients admitted to hospital between 1 April 2007 and 31 March 2017 with a primary or additional diagnosis of PHPT were identified from the hospital medical records. Hospital admission data were extracted from electronic medical records to identify patients with a discharge diagnostic code of ‘primary hyperparathyroidism’, ‘hyperparathyroidism other’, or ‘hyperparathyroidism unspecified’, or who had a procedural code of total parathyroidectomy or subtotal parathyroidectomy. Files were manually reviewed to confirm a diagnosis of PHPT (elevated calcium (>2.65 mmol/L) with elevated (>8.5 pmol/L), or normal (2–8.5 pmol/L), PTH level; or normal calcium with persistently elevated PTH level (normocalcaemic hyperparathyroidism)). Inclusion criteria were ≥18 years of age, admitted to hospital with a diagnosis of PHPT, and at least one DXA performed at our institution. Exclusion criteria were absence of a DXA performed at our institution, secondary or tertiary hyperparathyroidism (including chronic-kidney disease related hyperparathyroidism), medication-induced hyperparathyroidism, or insufficient information to establish a diagnosis of PHPT. The subgroup of individuals who had a DXA both before and at least 12 months after successful cure of PHPT by parathyroidectomy were included in the analysis of changes in bone architecture after parathyroidectomy. Inclusion criteria for this subgroup were parathyroidectomy with resolution of biochemical features of PHPT, DXA performed prior to or within 3 months of parathyroidectomy, and a second DXA at least 12 months post-operation. Individuals who were taking antiresorptive medications were included in all initial analyses. However, a second analysis was performed after excluding these individuals.

### 2.2. Outcome Measures

Demographic data and clinical history were extracted from medical records. Fragility fractures were defined as occurring from standing height and were confirmed on radiology. Clinical and non-clinical VF were included, and fractures of metacarpals, metatarsals, and phalanges were excluded. Anatomical pathology reports for histology and parathyroid gland weight were reviewed if parathyroidectomy was performed. Biochemistry at diagnosis included calcium adjusted for albumin (Ca), phosphate (PO_4_), alkaline phosphatase (ALP), and creatinine (Cr) levels assessed by automated analysers. Further, 25-OH-Vitamin D was analysed via chemiluminescence immunoassay (CLIA) (Liaison XL; DiaSorin, Saluggia, Italy), and intact PTH was analysed via CLIA (ADVIA Centaur; Siemens Healthcare, Victoria, Australia).

BMD at the LS, femoral neck (FN) and total hip (TH) was evaluated on Hologic 4500A densitometer (Hologic Inc., Bedford, MA, USA), which has a coefficient of variation of 1.00%, and least significant change of 0.00806 g/cm^2^ at the LS, 0.0104 g/cm^2^ and the FN, and 0.00963 g/cm^2^ at the TH. For the determination of FN and TH, the left side was used, except if individuals had a hip prosthesis, in which case the right was used, as is convention at our institution. T scores were derived using the manufacturer’s reference data. Where multiple DXA were available, the scan performed closest to diagnosis was used for analysis. Osteoporosis was defined as per World Health Organisation criteria as T score ≤ −2.5, and osteopenia as T score between −1.0 and −2.5. TBS was derived from LS DXA images retrospectively using TBS iNsight software version 3.0.2.0 (Medimaps; Geneva, Switzerland), and classified as degraded (TBS ≤ 1.20), partially degraded (TBS 1.21–1.34), and normal (TBS ≥ 1.35) [32].

The primary outcome was the TBS at the LS in adults with PHPT, compared to BMD, to describe bone quality, incorporating both the percentage of patients with normal or abnormal (osteoporosis/degraded) bone, and the ability to predict fragility fractures at diagnosis. The secondary outcome was to examine TBS and BMD at least 12 months after successful parathyroidectomy.

### 2.3. Statistical Analysis

Categorical variables are displayed as counts (%) and continuous variables as mean ± SD or median (Q1, Q3). To predict the incidence of one or more factures, we used logistic regression. We fitted multiple models using the diagnostic measures separately, combined, and with/without the demographic covariates age, sex, and body mass index (BMI). The predictive performance of each model was visualised by plotting the smoothed receiver operating characteristic (ROC) curve using the predicted probability of fractures versus the observed incidence of fractures. Predictive accuracy was summarised using the area under the curve (AUC), and 95% confidence intervals (CI) for the AUC were obtained by bootstrapping the combined procedure of fitting a logistic regression model and obtaining AUC from its predictions, using 5000 bootstrap replications. Descriptive, and not comparative statistics were used to compare BMD and TBS pre- and post- parathyroidectomy, due to the small sample size. Data were analysed with the R statistical program version 3.6.1 (R Core Team. Vienna, Austria), and a *p* value of <0.05 was considered significant.

## 3. Results

**Figure 1:**
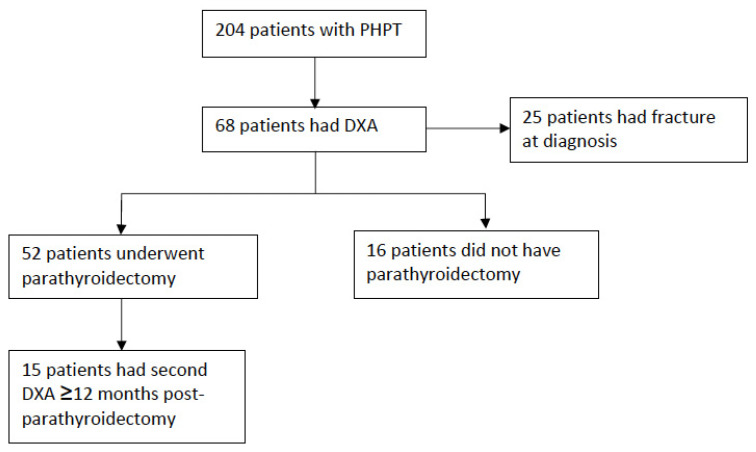
Study cohort.

A total of 204 patients were admitted with a diagnosis of PHPT, 68 of whom had DXA measurement. The included study cohort is displayed in Figure 1. Demographic and biochemical data at diagnosis are displayed in Table 1. Six individuals had normocalcaemic hyperparathyroidism. Ten individuals were on antiresorptive therapy (six oral bisphosphonates, three zoledronic acid, one denosumab). Data on other medications were not available.

### 3.1. Classificaion of Bone Healh Using TBS Compared to BMD

BMD and TBS results are displayed in Table 1. The percentage of individuals classified as osteoporosis or osteopenia on BMD, and as degraded or partially degraded on TBS, are displayed in Table 2 and Table 3. Combining BMD and TBS, only two individuals had normal bone architecture.

Of 24 individuals with normal LS BMD, 10 (41.7%) individuals had degraded TBS and 11 (45.8%) individuals had partially degraded TBS. Of the eight individuals with normal BMD at all sites, two (25.0%) individuals had degraded TBS and four (50.0%) individuals had partially degraded TBS. Of the five individuals with normal TBS, one (20%) had osteoporosis, two (40%) had osteopenia, and two (40%) had normal BMD.

### 3.2. TBS Compared to BMD for Predicting Fracture

**Figure 2:**
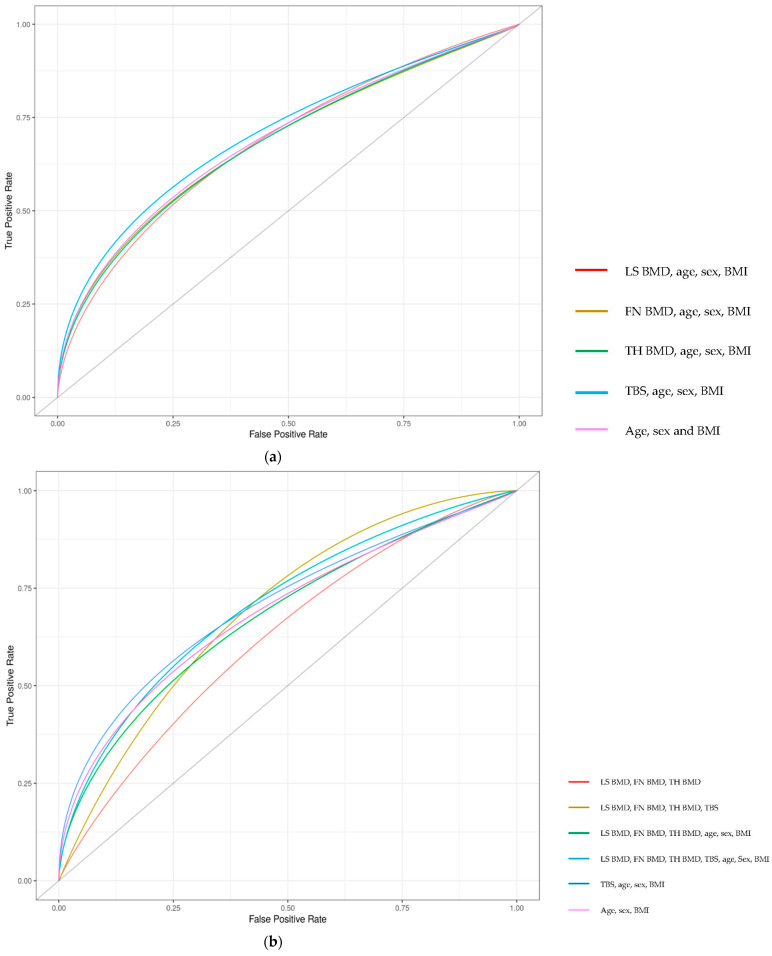
ROC for fracture prediction using BMD and TBS. (**a**): BMD of different regions and TBS for predicting fractures. (**b**): Combination of BMD regions with and without TBS for predicting fractures. LS: lumbar spine; BMD: bone mineral density; FN: femoral neck; TH: total hip; TBS: trabecular bone score; BMI: body mass index.

Among 68 individuals, 25 had a total of 42 prevalent fractures at diagnosis. ROC for BMD and TBS, adjusted for age, gender, and BMI as predicting fractures are displayed in Figure 2a. The AUC (95% CI) for predicting fractures was 0.683 (0.568, 0.840) for LS BMD, 0.683 (0.578, 0.845) for FN BMD, 0.682 (0.578, 0.831) for TH BMD, and 0.706 (0.592, 0.851) using TBS. The combination of the three BMD (LS, FN or TH) had an AUC of 0.678 (0.617, 0.870), and adding TBS, the AUC was 0.707 (0.647, 0.894) (Figure 2b).

### 3.3. Changes in BMD and TBS after Parathyroidectomy

Among 68 individuals, 52 underwent surgery for parathyroid adenoma resection with resolution of PHPT. The cohort who underwent surgery were older, had lower Ca and PTH levels, and more prevalent fractures than the cohort who did not undergo surgery (Table 1). Moreover, 15 of the 52 had DXA available prior to or within three months of operation, and at least 12 months post-operation, for comparison. The mean time of DXA pre-operation was 13.3 ± 23.3 months and post-operation was 40.7 ± 26.3 months. A comparison of results pre and post operation is displayed in Table 4. The BMD post-operation improved at all sites, and the TBS was reduced, although the absolute change was small. Exclusion of the two individuals previously treated with antiresorptive therapy, or the three individuals whose baseline DXA was within three months post-operation, did not alter results.

## 4. Discussion

This study has several key findings. We found degraded trabecular architecture determined by TBS in the majority of adults with PHPT, despite less than half having osteoporosis based on areal BMD by DXA. There was a correlation between TBS and prevalent fragility fractures at diagnosis in PHPT, and a trend towards improved fracture prediction using TBS. TBS reduced post-parathyroidectomy, although the magnitude of change was small.

Some previous studies have shown similar findings of degraded trabecular architecture in PHPT, despite osteopenia or normal BMD on DXA [24,33]. In contrast, the largest study to date of 123 individuals with PHPT did not find any additional benefit of TBS compared to BMD when assessing bone health [34]. The cohort examined was younger than our cohort, with fewer fractures and higher mean TBS of 1.25. It is unclear whether the duration or severity of PHPT, or other factors, may lead to such discrepancies.

At the LS, a site of predominantly trabecular bone, areal BMD, measured by DXA, is frequently preserved in PHPT [4,5]. In our study, over one third of individuals had normal BMD at the LS. Iliac crest biopsies have also documented preserved trabecular bone in PHPT [6]. However, studies comparing cadaver biopsies from the LS and iliac crest have shown significant discrepancy between the two sites, suggesting that iliac crest histomorphometry may not accurately reflect lumbar spine microarchitecture [35,36].

Over 90% of our cohort with PHPT had degraded or partially degraded bone architecture on TBS. This is consistent with HRpQCT findings, which demonstrate reduced trabecular volumetric BMD and decreased trabecular number, thickness, and connectivity in individuals with PHPT [10,11,12]. The discrepancy between DXA and iliac crest biopsies and VF incidence in PHPT suggests that HRpQCT, and now TBS, are more reflective of underlying bone abnormality in PHPT.

Individuals with even mild PHPT have increased rates of vertebral, distal forearm, rib, and pelvic fractures compared to controls [7,8]. TBS has been shown to be a stronger predicter of VF in PHPT than BMD at the LS, FN, or TH by DXA, although combinations of these parameters may be even more accurate [25]. Fewer studies have included non-vertebral fractures. One recent study compared TBS to BMD in predicting all prevalent fractures [33]. Similar to our findings, there was no difference in the performance of LS BMD, FN BMD, TH BMD, or TBS to predict fractures. In contrast to our study, when the combined model of any BMD site was compared to any BMD site with TBS, the model including TBS had better predictive value. Our results have shown a trend towards better fracture prediction using TBS. However, a larger sample size is required to determine the significance of this.

Parathyroidectomy results in improved BMD and reduces fracture risk in PHPT [37,38,39]. Despite us demonstrating abnormal bone architecture in more patients using TBS than BMD, and TBS correlating with fragility fractures, our cohort did not show improvement in TBS at least 12 months after parathyroidectomy. This discrepancy may be due to the small sample size or short duration of follow-up, as well as differences in the time to recovery of the trabecular bone. Previous studies analysing TBS before and 6–24 months after parathyroidectomy have had mixed results. The largest study examined 37 individuals with PHPT and showed a small but significant improvement in TBS six months after parathyroidectomy. In this study, the TBS of 1.26 was partially degraded, suggesting milder disease compared to our cohort [26]. Other studies have shown no improvement in TBS, despite the variable duration of disease prior to surgery from <12 months to 7.4 years [29,31]. A sub-analysis comparing gender differences found no significant improvement in TBS after parathyroidectomy in females, but a marked improvement in males [27]. However, this has not been confirmed in other studies. Analysis of the effects of BMI also found no differences in TBS changes between obese and non-obese individuals after surgery [30]. Further studies are warranted to examine other factors that may affect TBS changes post-parathyroidectomy.

Limitations of this study include that it is retrospective. Therefore, the timing of DXA and biochemistry in relation to diagnosis and parathyroidectomy was not standardised. The retrospective nature of our study meant that we were unable to prospectively follow patients to determine the risk of new fractures over time. The small sample size led to wide CIs, and we elected not to use comparative statistics for this reason. A repeat DXA after parathyroidectomy was only available in a small proportion of our subjects and was performed at various time points after surgery. The study was from a single centre so may not be generalisable to other settings. The cohort was predominantly older females, who have a greater risk of osteoporosis and fractures. However, this is reflective of the population predominantly affected by PHPT [1].

## 5. Conclusions

In this cohort of adults with PHPT, a majority had abnormal bone microarchitecture based on TBS, independent of BMD, suggesting that TBS provides complementary information about bone health in this population. In our cohort, the addition of TBS to BMD did not improve fracture prediction, and TBS did not improve after parathyroidectomy. However, these findings may be limited by the small sample size. Further prospective studies with larger sample sizes will help to determine the role of TBS in stratifying fracture risk in this disorder and monitoring changes in bone structure after parathyroidectomy.

## Figures and Tables

**Table 1:** Demographic data.

Parameter	Whole Cohort (*n* = 68)	Underwent Parathyroidectomy (*n* = 52)	No Parathyroidectomy (*n* = 16)
Age (years)	65.3 ± 17.9	61.8 ± 17.6	76.6 ± 14.3
Female	54 (79.4)	41 (78.8)	12 (75.0)
Caucasian ethnicity	54 (79.4)	39 (75)	14 (87.5)
BMI (kg/m^2^)	29.0 ± 7.87	29.1 ± 8.7	28.9 ± 4.7
Antiresorptive therapy	10 (14.7)	6 (11.5)	4 (25)
Fracture number at diagnosis ^∧^			
0	36 (52.9)	30 (57.7)	6 (37.5)
1	15 (22.1)	9 (17.3)	6 (37.5)
>1	10 (14.7)	7 (13.5)	3 (18.8)
Fracture type			
VF	12 (17.6)	8 (15.4)	4 (25.0)
Hip fracture	3 (4.4)	1 (1.9)	2 (12.5)
Upper limb	9 (13.2)	6 (11.5)	3 (18.8)
cCa (mmol/L)	2.83 ± 0.26	2.86 ± 0.28	2.73 ± 0.15
PTH (pmol/L)	26.2 ± 37.2	30.0 ± 41.7	13.8 ± 6.7
25(OH)Vit D (nmol/L) ^∨^	52.0 ± 18.8	52.1 ± 16.8	52.0 ± 24.8
Deficient <50 nmol/L	31 (46.3)	23 (44.2)	8 (50.0)
Severely deficient, <30 nmol/L	9 (13.4)	6 (11.5)	3 (18.8)
Cr (umol/L, mean) ^≈^	79.4 ± 34.0	80.3 ± 35.4	76.5 ± 23.5
eGFR (mL/min/1.73 m^2^, mean) ^≈^	70.5 ± 19.4	70.5 ± 20.1	70.7 ± 17.3
LS BMD	0.890 ± 0.184	0.872 ± 0.179	0.948 ± 0.193
LS T-Score	−1.51 ± 1.63	−1.66 ± 1.58	−1.00 ± 1.71
FN BMD	0.630 ± 0.126	0.634 ± 0.127	0.615 ± 0.128
FN T-Score	−2.07 ± 0.99	−2.04 ± 0.97	−2.18 ± 1.06
TH BMD	0.794 ± 0.163	0.801 ± 0.166	0.771 ± 0.157
TH T-Score	−1.32 ± 1.17	−1.25 ± 1.18	−1.54 ± 1.14
TBS	1.19 ± 0.12	1.19 ± 0.12	1.18 ± 0.12

Data expressed as mean ± SD or *n* (%). BMI: body mass index as kg/m^2^; VF: vertebral fracture; cCa: corrected calcium; PTH: parathyroid hormone; 25(OH)Vit D: 25-hydroxy-vitamin D3; Cr: creatinine; eGFR: estimated glomerular filtration rate; LS: lumbar spine; BMD: bone mineral density; FN: femoral neck; TH: total hip; TBS: trabecular bone score. Missing data for: ^∧^ 7 individuals; ^∨^ 1 individual; ^≈^ 2 individuals.

**Table 2:** Classification of bone health using BMD.

	LS BMD	FN BMD ^∧^	TH BMD	Any BMD
Osteoporosis	14 (20.6)	25 (37.3)	9 (13.2)	29 (42.6)
Osteopenia	30 (44.1)	31 (46.3)	33 (48.5)	31 (45.6)
Normal	24 (35.3)	11 (16.4)	26 (38.3)	8 (11.8)

Data expressed as *n* (%). BMD: bone mineral density; LS: lumbar spine; FN: femoral neck; TH: total hip. ^∧^ Missing data in 1 individual.

**Table 3:** Classification of bone health using TBS.

Classification	TBS
Degraded	39 (57.4)
Partially degraded	24 (35.3)
Normal	5 (7.3)

Data expressed as *n* (%). TBS: trabecular bone score.

**Table 4:** Comparison of BMD and TBS pre- and post-parathyroidectomy.

Parameter	Pre-Parathyroidectomy	Post-Parathyroidectomy
LS BMD	0.839 (0.631, 0.888)	0.862 (0.713, 0.937)
LS T-Score	−2.3 (−4.2, −1.4)	−1.8 (−3.2, −1.0)
FN BMD	0.592 (0.505, 0.700)	0.624 (0.534, 0.739)
FN T-Score	−2.3 (−3.1, −1.4)	−2.0 (−3.1, −1.2)
TH BMD	0.831 (0.578, 0.966)	0.847 (0.658, 0.970)
TH T-Score	−0.9 (−2.6, −0.4)	−0.8 (−2.3, −0.2)
TBS	1.17 (1.05, 1.20)	1.13 (0.95, 1.21)

Data expressed as median (Q1, Q3). BMD: bone mineral density; TBS: trabecular bone score; LS: lumbar spine; FN: femoral neck; TH: total hip.

## Data Availability

Data are available on request from the corresponding author.
